# Structure-activity relationship and target investigation of 2-aryl quinolines with nematocidal activity

**DOI:** 10.1016/j.ijpddr.2024.100522

**Published:** 2024-01-23

**Authors:** Harrison T. Shanley, Aya C. Taki, Nghi Nguyen, Tao Wang, Joseph J. Byrne, Ching-Seng Ang, Michael G. Leeming, Shuai Nie, Nicholas Williamson, Yuanting Zheng, Neil D. Young, Pasi K. Korhonen, Andreas Hofmann, Bill C.H. Chang, Tim N.C. Wells, Cécile Häberli, Jennifer Keiser, Abdul Jabbar, Brad E. Sleebs, Robin B. Gasser

**Affiliations:** a Department of Veterinary Biosciences, Melbourne Veterinary School, Faculty of Science, The University of Melbourne, Parkville, Victoria, 3010, Australia; b Chemical Biology Division, Walter and Eliza Hall Institute of Medical Research, Parkville, Victoria, 3052, Australia; c Melbourne Mass Spectrometry and Proteomics Facility, The Bio21 Molecular Science and Biotechnology Institute, The University of Melbourne, Parkville, Victoria, 3010, Australia; d National Reference Centre for Authentic Food, Max Rubner-Institut, 95326, Kulmbach, Germany; e Medicines for Malaria Venture (MMV), 1215, Geneva, Switzerland; f Medical Parasitology and Infection Biology, Swiss Tropical and Public Health Institute, 4123, Allschwil, Switzerland; g University of Basel, 4001, Basel, Switzerland

**Keywords:** Anthelmintics, Drug discovery, *Haemonchus contortus*, Target identification, Thermal proteome profiling, *In silico* docking

## Abstract

Within the context of our anthelmintic discovery program, we recently identified and evaluated a quinoline derivative, called ABX464 or obefazimod, as a nematocidal candidate; synthesised a series of analogues which were assessed for activity against the free-living nematode *Caenorhabditis elegans*; and predicted compound-target relationships by thermal proteome profiling (TPP) and *in silico* docking. Here, we logically extended this work and critically evaluated the anthelmintic activity of ABX464 analogues on *Haemonchus contortus* (barber's pole worm) – a highly pathogenic nematode of ruminant livestock. First, we tested a series of 44 analogues on *H. contortus* (larvae and adults) to investigate the nematocidal pharmacophore of ABX464, and identified one compound with greater potency than the parent compound and showed moderate activity against a select number of other parasitic nematodes (including *Ancylostoma, Heligmosomoides* and *Strongyloides* species). Using TPP and *in silico* modelling studies, we predicted protein HCON_00074590 (a predicted aldo-keto reductase) as a target candidate for ABX464 in *H. contortus*. Future work aims to optimise this compound as a nematocidal candidate and investigate its pharmacokinetic properties. Overall, this study presents a first step toward the development of a new nematocide.

## Highlights

• ABX464, a quinoline molecule, displays anthelmintic activity against *H. contortus.*
• ABX464 and one particular analogue, 36, are also active against several parasitic nematodes.
• HCON_00074590, an aldo-keto reductase, is identified as a nematode drug target.
• *In silico* docking predicts ligand interactions with *H. contortus* protein HCON_00074590.

## Graphical abstract

**Figure:**
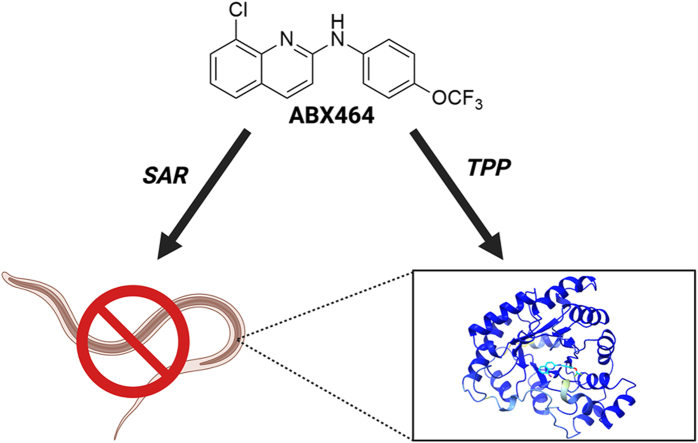

## 1 Introduction

Diseases (helminthiases) caused by gastrointestinal worms (helminths) continue to have a significant adverse impact on the health of both humans and animals, inflicting substantial socioeconomic losses worldwide (Casuli, 2021; Montresor et al., 2022; Shephard et al., 2022; World Health Organization, 2023). For example, in humans, soil-transmitted helminths cause diseases such as ascariasis, hookworm disease, strongyloidiasis and trichuriasis, which currently affect ∼1.5 billion people in poverty-stricken communities (Casuli 2021; Holland et al., 2022; World Health Organization, 2023). In animals, infections and diseases caused by gastrointestinal nematodes of the order Strongylida (strongylids), including species of *Haemonchus, Cooperia, Ostertagia*, *Teladorsagia* and *Trichostrongylus*, contribute to annual losses estimated at US$ 2.4 billion to the livestock industries in Australia and Europe, with the global animal-focussed antiparasitic market accounting for US$ 8 billion in sales (Charlier et al., 2021; Selzer and Epe, 2021; Shephard et al., 2022).

The control of these parasites is critical to alleviate these losses and disease problems. In human health, parasite control strategies focus on improving sanitation and hygiene (Campbell et al., 2014), and the use of mass drug administration to high-risk populations (World Health Organization, 2023). Parasite control in animals is best achieved through an integrated strategy, which includes sound management practices, built on knowledge and understanding of the epidemiological and climatic factors contributing to transmission, parasitism and disease (reviewed by Kahn and Woodgate, 2012; Maqbool et al., 2017), an effective anthelmintic treatment regimen, and, importantly, the monitoring of infections and infection intensity (prior to and/or after treatment) using diagnostic tools (Terrill et al., 2012; Kearney et al., 2016; Tinkler, 2020). Although vaccination to prevent gastrointestinal nematode infections would be preferred over the use of anthelmintic treatment, developing well-defined, recombinant vaccines against parasitic nematodes has been extremely challenging (reviewed by Britton et al., 2020, Diemert et al., 2018, Ehsan et al., 2020, Zawawi and Else, 2020). The only commercially available, ‘dead’ vaccine, Barbervax®, for use in animals induces protection against haemonchosis (caused by *Haemonchus contortus*) in small ruminants. However, protection has been reported to be variable in distinct host species (e.g., sheep *versus* goats; Meier et al., 2016; de Matos et al., 2017), between different age-groups (Kabagambe et al., 2000; Smith et al., 2001; Le Jambre et al., 2008; Bassetto et al., 2014; Smith, 2014), under differing levels of larval challenge (Kebeta et al., 2021), and additionally, vaccine ‘boosts’ are required to maintain protection (Smith et al., 2001; Bassetto et al., 2014; Smith, 2014).

Anthelmintic treatment remains an important part of most control campaigns. However, a heavy reliance on, and a misuse of, commercially available anthelmintics has led to the widespread development of drug resistance in parasitic nematodes of livestock animals (Sargison, 2016; Kotze and Prichard, 2016; Hodgkinson et al., 2019; Kaplan, 2020; Rose Vineer et al., 2020; Charlier et al., 2021; Charlier et al., 2022), with additional concerns of anthelmintic failure/resistance in humans (Vercruysse et al., 2011; Tinkler, 2020). For example, resistance in *H. contortus* to five (the amino-acetonitrile derivatives, benzimidazoles, imidazothiazoles, macrocyclic lactones and salicylanilides) of the six main drug classes available commercially (excluding derquantel) has been recorded (e.g., Le Jambre et al., 1979; Green et al., 1981; Rolfe et al., 1989; Le Jambre, 1993), with reports of monepantel resistance (Mederos et al., 2014) emerging five years following commercial release (Kaminsky et al., 2008). Issues such as these lend continual demand for novel anthelmintics whose modes of action differ from those that have been routinely, and often excessively, used and have induced widespread resistance.

New anthelmintic compounds have been identified using advanced, motility- and developmental inhibition-based whole-organism, phenotypic screening (reviewed by Herath et al., 2022). Early-stage drug discovery has been aided further by advances in mass spectrometry-based proteomics (e.g., Lomenick et al., 2009; Strickland et al., 2013; Savitski et al., 2014), which have been used to identify or infer drug-target interactions (reviewed by Hong and Lee, 2020; Ha et al., 2021). Moreover, some nematodes have been established as model organisms – including the well-defined and versatile free-living *Caenorhabditis elegans* (Brenner, 1974; Harris et al., 2020), and the highly pathogenic, blood-feeding ruminant parasite *H. contortus* (see Veglia, 1915). Despite biological differences, genomic, transcriptomic, proteomic and lipidomic investigations have revealed biochemical and genetic similarities between *H. contortus* (see Wang et al., 2018; Wang et al., 2019a; Wang et al., 2019b; Wang et al., 2020; Doyle et al., 2020), *C. elegans* (see Harris et al., 2020) and other parasitic nematodes of humans and animals, such as related species of *Ancylostoma* (Schwarz et al., 2015), *Necator* (Tang et al., 2014) and *Teladorsagia* (Hassan et al., 2023), indicating a potential to discover nematocides with a relatively broad spectrum of activity. Indeed, many of the current, commercially-available anthelmintics display activity in multiple nematode species, indicating that the discovery of broad-spectrum nematocides is achievable.

**Fig. 1:**
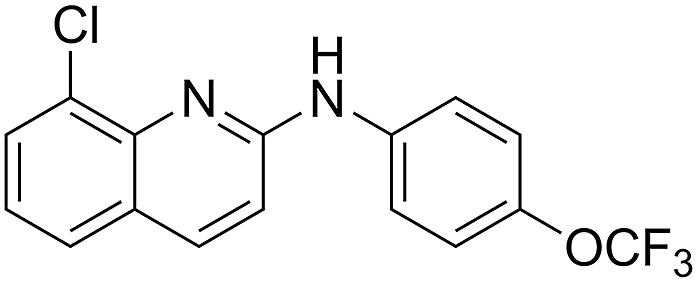
The chemical structure of ABX464.

To contribute to the discovery of new anthelmintic entities, we recently evaluated a quinoline derivative (ABX464, called obefazimod; Abivax, France, Fig. 1) (Campos et al., 2015; Vautrin et al., 2019; Tazi et al., 2020; Vermeire et al., 2022) identified in a phenotypic screen of the Pandemic Response Box (curated by the Medicines for Malaria Venture, Switzerland; cf. Shanley et al., 2022; Nick et al., 2022), for its nematocidal activity on *C. elegans* and *H. contortus.* Recently, ABX464 successfully underwent phase II clinical trials in the treatment of human ulcerative colitis as an anti-inflammatory compound (Vermeire et al., 2022). In humans, ABX464 interacts with the cap binding complex (Campos et al., 2015; Vautrin et al., 2019) and selectively upregulates miRNA-124 (Campos et al., 2015; Vautrin et al., 2019), a crucial modulator of inflammation (Tazi et al., 2020). Thus, it was proposed that ABX464 might be repurposed as an anthelmintic candidate for use in animals. As such, we synthesised a series of analogues which were assessed for activity against *C. elegans* (see Shanley et al., 2024) in a motility-based assay (Taki et al., 2021a), gleaning insights into the nematocidal pharmacophore of ABX464. Furthermore, we used thermal proteome profiling (TPP) (Savitski et al., 2014; Franken et al., 2015; Reinhard et al., 2015; Becher et al., 2016; Perrin et al., 2020; Mateus et al., 2022; Taki et al., 2022) to investigate potential drug target candidates of ABX464 in *C. elegans*, identifying two proteins of interest – designated CRN-3 and LUC-7L3.

To explore the anthelmintic activity of ABX464 on a parasitic species, here, we tested a series of analogues (Shanley et al., 2024) on larvae of *H. contortus* to establish a structure-activity relationship (SAR). We further evaluated key derivatives of ABX464 on *H. contortus* adults and a panel of parasitic nematodes (Keiser et al., 2016; Keiser and Häberli, 2021), and proceeded to infer the target(s) of ABX464 in *H. contortus* using TPP and *in silico* docking.

## 2 Materials and methods

### 2.1 Chemistry

Details of the synthesis and structural characterisation of compounds were published by Shanley et al. (2024). In brief, ABX464 and 44 derivatives were synthesised *via* a number of synthetic pathways, purified (≥95 % purified identified *via* high-performance liquid chromatography) and characterised *via* ^1^H nuclear magnetic resonance (NMR), ^13^C NMR, ^19^F NMR (where applicable), mass spectrometry (MS) and high-resolution MS. The structures of ABX464 and the 44 derivatives are given in Additional File 1: Fig. S1.

### 2.2 Biological assays

#### 2.2.1 H. contortus larvae and adult procurement

*H. contortus* (Haecon-5 strain; cf. Schwarz et al., 2013) was produced in experimental sheep as described previously (Schwarz et al., 2013; Preston et al., 2015) and in accordance with the institutional animal ethics guidelines (permit no. 1714374; The University of Melbourne, Parkville, VIC, Australia). Helminth-free Merino sheep (six months of age; male) were orally inoculated with 7000 third-stage larvae (L3s) of *H. contortus*. Four weeks after inoculation, faecal samples were collected from sheep with patent *H. contortus* infection. These samples were incubated at 27 ^°^C and >90 % relative humidity for one week to yield L3s (Preston et al., 2015), which were then collected in tap water and allowed to migrate through two layers of nylon mesh (pore size: 20 μm; Rowe Scientific, Doveton, VIC, Australia) to remove debris. Clean L3s were stored in the dark at 11 ^°^C for up to six months (Preston et al., 2015).

Adult *H. contortus* were collected from the abomasa of sheep infected for 10 weeks, washed extensively with phosphate-buffered saline (PBS, pH 7.4) and subsequently in RPMI 1640 media supplemented with final concentrations of 2 mM L-glutamine, 100 IU/mL of penicillin, 100 μg/mL of streptomycin and 0.25 μg/mL of amphotericin B (Thermo Fisher Scientific, Scoresby, VIC, Australia; this supplemented RPMI was designated RPMI*). Female and male worms were collected and separated immediately prior to compound testing.

#### 2.2.2 H. contortus larvae preparation and dose-response assay

Immediately prior to screening, *H. contortus* L3s were artificially exsheathed *via* exposure to 0.15 % (*v*/*v*) sodium hypochlorite for 20 min at 38 ^°^C (Preston et al., 2015), achieving an exsheathment rate of 90 %. The larvae were then immediately washed five times with 50 mL of sterile physiological saline solution by centrifugation at 800×*g* (5 min) and resuspended (at a concentration of 300 xL3s per 50 μL) in sterile (autoclaved) lysogeny broth (LB; cf. Bertani, 1951; Taki et al., 2021b), supplemented with final concentrations of 100 IU/mL of penicillin, 100 μg/mL of streptomycin and 0.25 μg/mL of amphotericin B (Fungizone®, cat. No. 15240-062, Gibco, Thermo Fisher Scientific, Waltham, MA, USA); this supplemented LB was designated LB*.

The dose-response assay for *H. contortus* followed a well-established protocol (Taki et al., 2021b); it was employed to evaluate the potency of hit compounds against this nematode. Test compounds were assessed individually for an effect on the motility of xL3s (10-point, 2-fold serial dilution in LB*, 40 μM–0.16 μM). One compound, monepantel (prepared in the same manner as the test compounds), was used as a positive control. A solution of LB* was used as a negative control. The test compounds and positive control compound were arrayed in triplicate across individual flat-bottom 96-well microplates, with six wells on each plate containing the negative control. Added to each well were 300 xL3s of *H. contortus* in 50 μL of LB* to give a final volume of 100 μL. Plates were then placed in a CO_2_ incubator (10 % [*v*/*v*] CO_2_, 38 ^°^C, >90 % humidity; Forma, model no. 311, Thermo Fisher Scientific, USA). After 168 h of incubation, worm activity was captured using a WMicroTracker ONE unit. Over a period of 15 min, disturbance of an infrared beam in individual wells was recorded as a worm activity count. Raw ‘activity counts’ for each well were normalised to the negative-controls. The compound concentrations were log_10_-transformed and fitted using a variable slope four-parameter equation, using the ordinary least squares fit model, employing Prism (v.9.1.0 GraphPad Software, San Diego, CA, USA). Larval development was established at 168 h of incubation with compound, as described previously (Preston et al., 2015). The development inhibition and phenotypes of larvae were examined using a microscope (Preston et al., 2015).

#### 2.2.3 Assessment of the activity of selected compounds on H. contortus adults

The activity of ABX464 and three derivatives was assessed on adult female specimens of *H. contortus* in an established assay (Taki et al., 2020). The compound was added in triplicate to the wells of a 24-well plate (cat. no. 3524; Corning, USA) at a concentration of 40 μM in 500 μL of RPMI*. Two positive-control compounds, monepantel and moxidectin, and a negative control containing 1 % (*v*/*v*) DMSO only, were included in triplicates on the same plate. Three adult females were added to each of the triplicate wells containing either the test compound or the controls and placed in a CO_2_ incubator (10 % [*v*/*v*] CO_2_, 40 ^°^C, >90 % relative humidity) for 1 day. A video recording (30 s) of each well was taken at 3 h, 6 h, 12 h and 24 h during the total incubation period to assess the reduction in worm motility, which was scored as 3 (“good”), 2 (“low”), 1 (“very low”) or 0 (“no movement”; cf. Taki et al., 2020). For each test or control compound, the motility scores for each of the triplicate wells were calculated, normalised with reference to the negative control (100 % motility) and recorded as a percentage.

#### 2.2.4 Procurement of A. ceylanicum, N. americanus, He. polygyrus, T. muris and S. ratti

In accordance with institutional animal ethics guidelines and the regulations of Switzerland (permit no. 2070; Swiss Tropical and Public Health Institute), (three weeks of age; male) Syrian golden hamsters (Janvier Laboratories, Le Genest-Saint-Isle, France) were orally infected with 140 L3s of *A. ceylanicum* or 150 L3s of *N. americanus*; (three weeks of age; female) NMRI mice (Charles River Laboratories, Sulzfeld, Germany) were orally inoculated with 90 L3s of *He. polygyrus*; (three weeks of age; female) C57BL/6NRj mice (Janvier Laboratories) were orally inoculated with 200 embryonated eggs of *T. muris*; (three weeks of age; male) Wistar rats (Janvier Laboratories) were subcutaneously injected with 1300 L3s of *S. ratti* (Keiser and Häberli, 2021). Faeces (containing eggs) collected from animals infected with *A. ceylanicum*, *N. americanus*, *He. polygyrus* or *S. ratti* were incubated for 8–10 days (no light, 24 ^°^C, >90 % relative humidity) and, after purification, hatched L3 were used for drug assays; *S. ratti* L3s were then isolated and concentrated using the Baermann technique. Adults of *T. muris* were collected from the intestines of infected mice after 7 weeks. *A. ceylanicum* and *N. americanus* larvae were suspended in Hanks’ balanced salt solution (HBSS; Thermo Fisher Scientific); *He. polygyrus* in RPMI 1640 medium; *S. ratti* in PBS (pH 7.4); and *T. muris* in RPMI 1640 medium plus 5 % inactivated foetal calf serum (Bioconcept AG, Allschwil, Switzerland). Each medium was supplemented with 100 IU/mL of penicillin, 100 μg/mL of streptomycin and (except in the case of *S. ratti* and *T. muris*) 0.25 μg/mL of amphotericin B.

#### 2.2.5 Assessment of test compound activity on selected parasitic nematode species other than H. contortus

For each assay, ABX464 and one derivative were tested individually, in triplicate (larvae) or duplicate (adults), at a single concentration of 50 or 10 μM; appropriate medium plus the highest percentage of DMSO used in the assay was the negative control. Larvae of *A. ceylanicum*, *N. americanus*, *He. polygyrus* and *S. ratti* were dispensed into 96-well plates (density of 30–40 L3s per well) and incubated with compound (final volume of 250 μL in respective medium) in the dark at either 22 ^°^C (*A. ceylanicum*, *He. polygyrus, S. ratti*) or 37 ^°^C, 5 % (*v*/*v*) CO_2_, >90 % relative humidity (*N. americanus*). Following 72 h of incubation, 50–80 μL of hot water (80 ^°^C) was added to each well; larval death was then measured by counting motile L3s.

Adults of *T. muris* were dispensed into a 24-well plate (density of three adults per well) and incubated with compound (final volume of 2 mL in the appropriate medium), in the dark at 37 ^°^C, 5 % (*v*/*v*) CO_2_, >90 % relative humidity. Following 72 h of incubation, 500 μL of hot water (80 ^°^C) was added to each well; adult worms were inspected microscopically using a viability scale from 3 (“normal activity”) to 0 (“dead”) as previously described (Keiser et al., 2016).

#### 2.2.6 Thermal proteome profiling (TPP)

TPP was conducted essentially using an established five-step protocol (cf. Taki et al., 2022).

##### 2.2.6.1 Preparation of protein extracts from H. contortus

*H. contortus* (2,000,000 L3s) were exsheathed as per section 2.2.2, collected by centrifugation (2000×*g* for 5 min) and frozen at −80 ^°^C, following the removal of the supernatant. Subsequently, the frozen pellet was ground to a fine powder in liquid nitrogen using a mortar and pestle, transferred to a 10 mL tube, suspended in 3 mL ice-cold phosphate-buffered saline (pH 7.0) containing 0.5 % (*v*/*v*) nonyl phenoxypolyethoxylethanol (NP-40) and lysed by gentle aspiration/expulsion using a 5 mL sterile syringe with a 22-gauge needle. Subsequently, the supernatant was collected from this suspension following centrifugation at 20,000×*g* for 20 min at 4 ^°^C. The protein concentration in the supernatant was measured using a BCA Protein Assay Kit (Thermo Fisher Scientific, USA), adjusted to 2 mg/mL and divided into four 250 μL aliquots/replicates (each containing 500 μg protein).

##### 2.2.6.2 Incubation with compound (ABX464) and temperature profile

Of the four 250 μL aliquots of *H. contortus* proteins, two (i.e. test-samples) were each incubated with an equal volume of compound (ABX464 at 50 μM), and two (control-samples) with an equal volume of PBS (pH 7.0) for 30 min at 23 ^°^C. Each of the samples (containing 500 μL) was partitioned into 10 PCR tubes (50 μL each); individual pairs of test- and control-samples were simultaneously incubated in a thermal cycler (Applied Biosystems) at 10 distinct temperatures (37, 41, 44, 47, 50, 53, 56, 59, 63 and 67 ^°^C) for 3 min. Subsequently, all 40 tubes were centrifuged 20,000×*g* for 20 min at 4 ^°^C, and soluble proteins (i.e. from above the pellet) collected into fresh tubes (each containing 45 μL).

##### 2.2.6.3 In-solution digestion and isobaric stable isotope labelling of peptides

Proteins in aliquots (45 μL) of individual samples (n = 40) were denatured in 8 M urea for 30 min at 37 ^°^C and diluted to < 2 M urea using lysis buffer prior to processing for in-solution digestion (Ang et al., 2011). Samples were reduced with 10 mM tris (2-carboxyethyl) phosphine, alkylated with 55 mM iodoacetamide, followed by digestion with trypsin (Promega) at 37 ^°^C for 16 h. The trypsin-treated samples were acidified with 1.0 % (*v*/*v*) formic acid (FA) and purified using Oasis HLB cartridges (Waters; wash solvent, 0.1 % FA; elution solvent, 80 % acetonitrile (CH_3_CN) in 0.1 % FA). Then, proteins were labelled with tandem mass tags (TMTs) (Zecha et al., 2019). In brief, desalted peptides were resuspended in 50 mM triethylammonium bicarbonate (pH 8.5) and mixed with a TMT10plex reagent (Thermo Fisher Scientific, USA) that was dissolved in 41 μL of anhydrous CH_3_CN. The TMT-peptide mixture was incubated for 1 h at 25 ^°^C with gentle shaking. Subsequently, 3.2 μL of 5 % (*w*/*v*) hydroxylamine was added to the mixture and incubated for 15 min at 25 ^°^C with gentle shaking to quench the reaction. Labelled peptides were combined accordingly and then desalted on Oasis HLB cartridges (Waters; using wash solvent, 0.1 % FA; elution solvent, 80 % CH_3_CN in 0.1 % FA). Each mixed peptide sample was separated into eight fractions using the high pH reversed-phase peptide fractionation kit (Pierce), according to the manufacturer's protocol. All fractions were freeze-dried prior to resuspension in aqueous 2 % (*w*/*v*) CH_3_CN and 0.05 % (*w*/*v*) trifluoroacetic acid (TFA) before LC-MS/MS analysis.

##### 2.2.6.4 LC-MS/MS analysis, and protein identification/annotation

LC-MS/MS was performed on the Exploris 480 Orbitrap mass spectrometer (Thermo Fisher Scientific, USA). The LC system was equipped with an Acclaim Pepmap nano-trap column (Dinoex-C18, 100 Å, 75 μm × 2 cm) and an Acclaim Pepmap RSLC analytical column (Dinoex-C18, 100 Å, 75 μm–50 cm). The tryptic peptides were injected into the enrichment column at an isocratic flow of 5 μL/min of 2 % (*v*/*v*) CH_3_CN containing 0.05 % (*v*/*v*) TFA for 6 min, applied before the enrichment column was switched in-line with the analytical column. The eluents were 0.1 % (*v*/*v*) FA (solvent A) in water and 100 % (*v*/*v*) CH_3_CN in 0.1 % (*v*/*v*) FA (solvent B), both supplemented with 5 % DMSO. The gradient was at 300 nL/min from (i) 0–6 min, 3 % B; (ii) 6–7 min, 3–4 % B; (iii) 7–82 min, 4–25 % B; (iv) 82–86 min, 25–40 % B; (v) 86–87 min, 40–80 % B; (vi) 87–90 min, 80–3 % B; (vii) 90–90.1 min, 80–3 % B and equilibrated at 3 % B for 10 min before injecting the next sample. The Exploris 480 Orbitrap mass spectrometer was operated in the data-dependent mode, whereby full MS1 spectra were acquired in a positive mode, with spray voltage at 1.9 kV, source temperature at 275 ^°^C, MS1 at 120,000 resolution, normalised AGC target of 300 % and maximum IT time of 25 ms. The top 3 s method was used and selecting peptide ions with charge states of ≥2–7 and intensity thresholds of ≥ 5 × 10^3^ were isolated for MS/MS. The isolation window was set at 0.7 *m/z*, and precursors were fragmented using higher energy C-trap dissociation (HCD) at a normalised collision energy of 35, a resolution of 30,000 (TurboTMT activated), a normalised AGC target of 200 % and automated IT time.

Mass spectrometry data were processed using MaxQuant (v2.1.1.0) for the identification and quantification of peptides/proteins. Proteins were matched to those inferred from the reference genome (version 4) for *H. contortus* (Doyle et al., 2020). The MaxQuant default methods were used for reporter MS2 TMT based workflow. The TMT reagent was corrected for natural carbon isotopes and incomplete stable isotope incorporation. Fixed modifications of carbamidomethylation of cysteine. Trypsin/P was set as the protease with a maximum of 2 missed cleavages. Variable modifications are oxidation of methionine and acetylation of protein N-terminus. All quantitative values were normalised based on the weighted ratio to reference channel function to the 1st TMT reference channel (126C) made up of a pool of each sample The isobaric matching between runs feature to improve reporter ion-based quantitation was also turned on. Protein and PSM false discovery rates (FDR) were both set at < 0.01. Results are available *via* the PRIDE data repository (accession number: PXD046553).

##### 2.2.6.5 Data processing and analysis

The quantitative protein data produced by MaxQuant was taken for analysis in R (v4.1.2). Decoy proteins, contaminant proteins, proteins only identified by modified peptides, and proteins that were identified by less than 2 razor or unique peptides were removed. Corrected reporter ion intensities were then divided by the intensity of the 37 ^°^C channel. Due to the marked decrease in overall protein abundance with increasing temperature, protein abundance ratios were grouped by treatment temperature and subjected to quantile normalisation using limma (v3.50.0) (Ritchie et al., 2015). Proteins were filtered to retain only those with non-zero values for each sample, and these were taken for subsequent analysis.

Thermal profiles of quantified proteins were assessed using the package NPARC (v1.6.0) (Childs et al., 2019), which fits nonparametric models to the temperature profile data under null and alternative hypotheses; p-values were then calculated from F-statistics with empirically estimated degrees of freedom, as described in the NPARC package documentation (Perrin et al., 2020). Melting profiles were plotted and manually inspected for top ranking protein hits that were statistically significant (Benjamini-Hochberg-adjusted p-values were <0.05).

#### 2.2.7 In silico protein-ligand docking

The three-dimensional structure of HCON_00074590 (UniProt accession codes: A0A7I5E8V5) was accessed from the AlphaFold2 database (Jumper et al., 2021; Varadi et al., 2021), an advanced program which uses deep-learning to predict the three-dimensional structure of proteins from their primary amino acid sequences with high levels of confidence. To model interactions between the *H. contortus* protein and compound (ABX464), the AutoDock Vina tool software (Trott and Olson, 2010) was utilised; the protein model was prepared utilising the AutoDock tool prepare_receptor (energy minimisation of the protein structure), the ligand (sdf-format) was prepared using the prepare_ligand tool (addition of missing atoms, assignment of charges and optimisation of ligand geometry). The search space for binding cavities was defined as the ‘complete protein structure’. The resultant protein-ligand binding conformations were visualised using ChimeraX v1.6.1 (Pettersen et al., 2021) and assessed for Vina score. Each predicted ligand-protein model was further assessed for per-residue confidence scores, with binding interactions within low confidence cavities (pLDDT <70) being excluded. Utilising InterPro (Paysan-Lafosse et al., 2022), the binding cavity identified in each model was then assessed for any associations with active sites and/or domains. Binding models were ranked using a ‘Vina score’ – confidence levels of surrounding residues and associated active domains.

## 3 Results

### 3.1 Structure-activity relationship investigation

#### 3.1.1 Analogue synthesis and structure-activity investigation of the aniline moiety

ABX464 (**1**) and all analogues were previously synthesised and structurally characterised (Shanley et al., 2024). The IC_50_ of ABX464 against *H. contortus* exsheathed third-stage larvae (xL3s) after 168 h of incubation with compound was first determined to be 6.0 μM, with a maximum motility inhibition (MMI) of 81 % (Additional File 1: Fig. S2). To investigate how altering the aniline moiety would impact activity, we first tested a *des-p-*trifluoromethoxy derivative (**2**), which displayed a reduced activity (IC_50_ = 16 μM; MMI = 88 %) (Table 1). Further testing of a *para* chloro (**6**, IC_50_ = 10 μM; MMI = 82 %) and a *para* fluoro (**7**, IC_50_ = 10 μM; MMI = 100 %) derivative found they had similar potency. In contrast, a *p*-methoxy derivative (**4**, IC_50_ = 8.5 μM; MMI = 86 %) was equipotent, whereas a methyl (**5**, IC_50_ = 14 μM; MMI = 82 %) derivative was slightly less active. A *p*-trifluoromethyl derivative (**25**, IC_50_ = 3.0 μM; MMI = 78 %, Additional File 1: Fig. S2) was found to be more active than the parent compound (**1**), whereas a nitrile (**3**), phenyl (**8**) or hydroxy (**33**) group all lost activity (IC_50_ ≥ 40 μM). Taken together, these results imply that activity at the *para* position may prefer bulkier, hydrophobic substituents (e.g., trifluoromethoxy, methoxy or chloro). Possibly, this position points towards a pocket able to accommodate these groups within a possible protein target. However, the loss of activity when a phenyl group was introduced suggests that space for extension within this pocket is limited. Possibly, the electron-withdrawing properties of a functional group at the *para* position may enhance activity, as in the case of compound **25**.

**Table 1:**
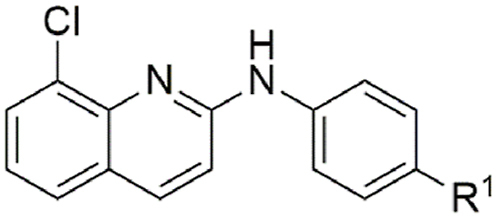
Activity of *para* substituted aniline ABX464 analogues on larvae of *H. contortus* after 168 h of incubation.

Compound	R^1^	Motility of larvae IC_50_ (SD) μM^a^
**1**	-OCF_3_	6.0 (1.8)
**2**	-H	16 (4.2)
**3**	-CN	>40
**4**	-OMe	8.5 (1.6)
**5**	-Me	14 (0.3)
**6**	-Cl	10 (0.3)
**7**	-F	10 (2.7)
**8**	-Ph	>40
**25**	-CF_3_	3.0 (0.9)
**33**	-OH	>40
Monepantel	N/A	0.08 (0.03)

a IC_50_ calculated from three independent assays in triplicate.

A series of *meta* mono-substituted derivatives (Table 2) were also investigated. Notably, the introduction of a *meta*-trifluoromethoxy (**9**, IC_50_ = 15 μM; MMI = 69 %), trifluoromethyl (**10**, IC_50_ = 4.9 μM; MMI = 64 %), or phenyl (**16**, IC_50_ = 4.6 μM; MMI = 64 %) functional groups led to compounds with the same activity as **1** but with reduced MMIs. In contrast, the *meta* methoxy (**12**), methyl (**13**), chloro (**14**) and fluoro (**15**) analogues retained activity (IC_50_ = 8.2 μM–20 μM), displaying a high MMI (84–100 %). It is possible that the differences in the MMI are a reflection of hydrophobicity, whereby hydrophobic functional groups are less tolerated than their less hydrophobic counterparts. However, inclusion of a *meta*-nitrile functional group (**11**) lost activity (≥40 μM), implying that polar substituents were not tolerated. In contrast, all substitutions made at the *ortho* position (**17**–**20**, **26**–**28**, Table 3), in addition to the incorporation of an endocyclic nitrogen (**21** and **30**), produced inactive (IC_50_ ≥ 40 μM) compounds. Additionally, a number of *para* and *meta* disubstituted derivatives (**22**, **23**, **24** and **31**, Table 4; Additional File 1: Fig. S2) were also tested for activity; however, only the 3,4-dichloro derivative **24** had activity, displaying an IC_50_ of 5.3 μM and MMI of 95 %.

**Table 2:**
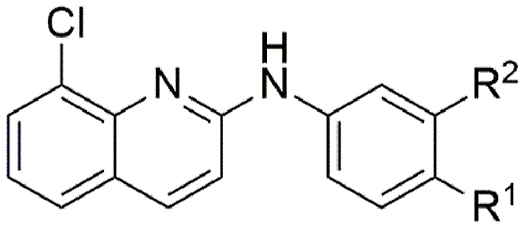
Activity of *meta* substituted aniline ABX464 analogues on larvae of *H. contortus* after incubation for 168 h.

Compound	R^1^(a)	R^2^(a)	Motility of larvae IC_50_ (SD) μM^b^
**1**	-OCF_3_	–	6.0 (1.8)
**9**	–	-OCF_3_	15 (5.1)
**10**	–	-CF_3_	4.9 (1.8)
**11**	–	-CN	>40
**12**	–	-OMe	8.2 (2.8)
**13**	–	-Me	20 (9.3)
**14**	–	-Cl	9.4 (0.07)
**15**	–	-F	12 (2.5)
**16**	–	-Ph	4.6 (2.9)
Monepantel	N/A	N/A	0.08 (0.03)

a A dashed line (‘ - ‘) indicates a –H atom.

b IC_50_ calculated from three independent assays in triplicate.

**Table 3:**
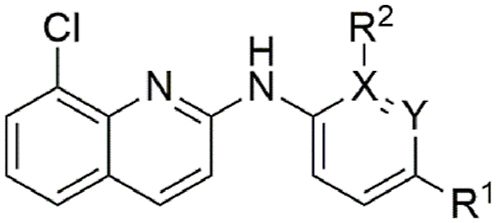
Activity of *ortho* substituted aniline and endocyclic nitrogen ABX464 analogues on larvae of *H. contortus* after incubation for 168 h.

Compound	R^1^(a)	R^2^(a)	X^b^	Y^b^	Motility of larvae IC_50_ (SD) μM^c^
**1**	-OCF_3_	–	–	–	6.0 (1.8)
**17**	–	-Cl	–	–	>40
**18**	–	-OMe	–	–	>40
**19**	–	-F	–	–	>40
**20**	–	-CN	–	–	>40
**21**	-CF_3_	–	–	N	>40
**26**	–	-OCF_3_	–	–	>40
**27**	–	-Ph	–	–	>40
**28**	–	-CF_3_	–	–	>40
**30**	-CF_3_	–	N	–	>40
Monepantel	N/A	N/A	N/A	N/A	0.08 (0.03)

a A dashed line (‘ - ‘) indicates a –H atom.

b A dashed line (‘ - ‘) indicates a –CH group.

c IC_50_ calculated from three independent assays in triplicate.

**Table 4:**
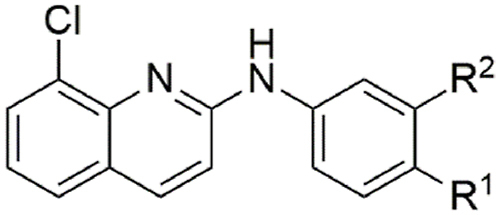
Activity of di-substituted ABX464 analogues on larvae of *H. contortus* after incubation for 168 h.

Compound	R^1^	R^2^	Motility of larvae IC_50_ (SD) μM^a^
**1**	-OCF_3_	-H	6.0 (1.8)
**22**	-OCF_3_	-Cl	>40
**23**	-Cl	-CF_3_	>40
**24**	-Cl	-Cl	5.3 (0.7)
**31**	-CF_3_	-CF_3_	>40
Monepantel	N/A	N/A	0.08 (0.03)

a IC_50_ calculated from three independent assays in triplicate.

Finally, N-methylation of the amine linker (**29**, Table 5) displayed reduced activity (IC_50_ = 13 μM; MMI = 58 %) compared with the parent molecule, whereas the replacement of the secondary amine with an oxygen atom (**32**) led to a loss of activity (IC_50_ > 40 μM), possibly implying the importance of the –NH hydrogen bond donor for activity.

**Table 5:**
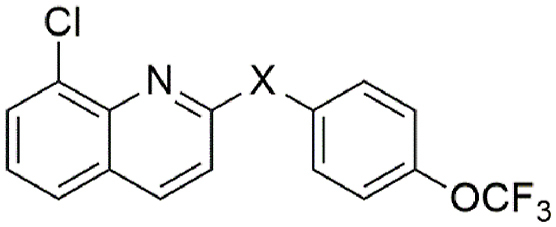
Activity of N-substituted ABX464 analogues on larvae of *H. contortus* after incubation for 168 h.

Compound	X	Motility of larvae IC_50_ (SD) μM^a^
**1**	NH	6.0 (1.8)
**29**	NMe	13 (6.1)
**32**	O	>40
Monepantel	N/A	0.08 (0.03)

a IC_50_ calculated from three independent assays in triplicate.

#### 3.1.2 Structure-activity investigation of the quinoline moiety

To investigate how alterations to the quinoline moiety of ABX464 affected activity (Table 6), we tested a *des*-chloro derivative (**34**, IC_50_ = 24 μM; MMI = 78 %) which led to a reduction in activity. Further investigation found that the 6- (**35**) and 4-chloro (**37**) analogues displayed a slightly lower activity (IC_50_ = 9.8 and17 μM, MMI = 75 and88 %, respectively) than the original compound, whereas a 3- chloro derivative, **38**, lost activity (IC_50_ > 40 μM). In contrast, the 5-chloro derivative (**36**) slightly gained activity, displaying an IC_50_ of 4.3 μM whilst retaining a MMI of 100 % (Additional File 1: Fig. S2). Replacement of the 8-chloro functional group with an 8-methoxy (**39**) group gave an equipotent compound (IC_50_ = 9.8 μM; MMI = 78 %), whereas the 8-methyl derivative (**40**) showed a reduction in activity (IC_50_ = 18 μM; MMI = 97 %). Both the 8-fluoro (**41**) and 8-bromo (**42**) derivatives retained equipotent activity (IC_50_'s of 8.4 and 5.2 μM, respectively). Finally, neither the 8-phenyl (**43**) or 8-nitrile (**44**) derivatives, nor a replacement of the quinoline structure with a pyridine motif (**45**) displayed activity (IC_50_ > 40 μM).

**Table 6:**
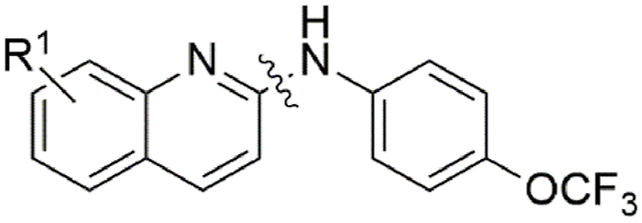
Activity quinoline substituted ABX464 analogues on larvae of *H. contortus* after incubation for 168 h.

Compound	R^1^	Motility of larvae IC_50_ (SD) μM^a^
**1**	8-Cl	6.0 (1.8)
**34**	8-H	24 (13)
**35**	6-Cl	9.8 (2.3)
**36**	5-Cl	4.3 (1)
**37**	4-Cl	17 (9.3)
**38**	3-Cl	>40
**39**	8-OMe	9.8 (4.8)
**40**	8-Me	18 (1.4)
**41**	8-F	8.1 (1.5)
**42**	8-Br	5.2 (2.2)
**43**	8-Ph	>40
**44**	8-CN	>40
**45**^b^	(2-pyridyl)	>40
Monepantel	N/A	0.08 (0.03)

a IC_50_ calculated from three independent assays in triplicate.

b In compound 45, the quinoline scaffold has been replaced with a pyridine.

### 3.2 Three analogues significantly inhibited H. contortus larval development and/or adult female motility

After an incubation for 168 h, ABX464 inhibited larval development (Table 7) with an IC_50_ of 3.5 μM. In contrast, three compounds, **24**, **25** and **36** (IC_50_'s of 2.3, 3.9 and 4.1 μM respectively) showed an equal or slightly pronounced inhibition of larval development (Additional File 1: Fig. S3). Furthermore, all four compounds (including ABX464) induced an abnormal curved (*Cur*) phenotype at a concentration of 40 μM. We further assessed ABX464 and these three key derivatives (40 μM concentration) for the motility inhibition of *H. contortus* adult females after 24 h of incubation (Table 7). ABX464 reduced adult female motility by 73 %, compound **24** by 20 %, compound **25** by 0 %, and compound **36** by 87 %. In contrast, the monepantel and moxidectin controls reduced adult female motility by 100 % and 60 %, respectively, after 24 h of incubation (Additional File 1: Fig. S4).

**Table 7:** Activity of ABX464 key analogues on *H. contortus*, *A. ceylanicum*, *Necator americanus*, *He. polygyrus*, *S. ratti* and *T. muris*.

Compound	*H. contortus*			Worm death in % (SD)^d^^,^^e^					
	Larval inhibition IC_50_ (SD) μM^a^	Larval phenotype^a^^,^^b^	Adult female motility reduction (%)^c^	*A. ceylanicum*	*N. americanus*	*He. polygyrus*		*S. ratti*	*T. muris*
						50 μM	10 μM		
**ABX464 (1)**	3.5	*Cur*	73	43 (15)	13 (3.8)	15 (0.7)	5.2 (5.5)	64 (3.3)	25 (0)
**24**	2.3	*Cur*	20	–	–	–	–	–	–
**25**	3.9	*Cur*	0	–	–	–	–	–	–
**36**	4.1	*Cur*	87	25 (0.2)	9.7 (2)	59 (6.9)	26 (8.7)	44 (0.7)	13 (0)
Monepantel	0.27	*Coi*	100	–	–	–	–	–	–

a Measured after 168 h of incubation.

b Curved, *cur*; coiled, *coi*.

c Measured after 24 h of incubation.

d Measured after 72 h of incubation. Unless otherwise stated, compounds were tested at a concentration of 10 μM.

e *A. ceylanicum, N. americanus, He. polygyrus* and *S. ratti* are third-stage larvae; *T. muris* are adult (male and female) worms.

### 3.3 ABX464 and compound 36 display activity against other nematode species

Given that both ABX464 and compound **36** had anthelmintic activity on *H. contortus* larvae and adult worms, and were previously reported to have activity on *C. elegans* larvae, we investigated these compounds further for nematocidal activity on the related parasitic (clade V) strongylid nematodes (third-stage larvae, L3), *Ancylostoma ceylanicum, Necator americanus* and *Heligmosomoides polygyrus*, as well as the distantly related nematodes *Strongyloides ratti* (L3s, clade IV) and *Trichuris muris* (adults, clade I).

In short, after 72 h of incubation with a single concentration (50 or 10 μM) of compound (Table 7), ABX464 inhibited *He. polygyrus* by 15 % (50 μM) and 5.2 % (10 μM), *N. americanus* by 13 % (10 μM), *A. ceylanicum* by 43 % (10 μM), *S. ratti* by 64 % (10 μM) and *T. muris* by 25 % (10 μM). In comparison, compound **36** inhibited *He. polygyrus* by 59 % (50 μM) and 26 % (10 μM), *N. americanus* by 9.7 %, *A. ceylanicum* by 25 %, *S. ratti* by 45 % and *T. muris* by 12.5 %.

### 3.4 Evidence of multiple ABX464 protein targets in H. contortus

To investigate possible protein targets of ABX464 in *H. contortus*, a protein lysate of xL3s of this species was incubated with 50 μM of ABX464 and then subjected to TPP across a gradient of 37 °C–67 °C (Taki et al., 2022; Shanley et al., 2024), to identify proteins with altered stability in the presence of ABX464.

**Fig. 2:**
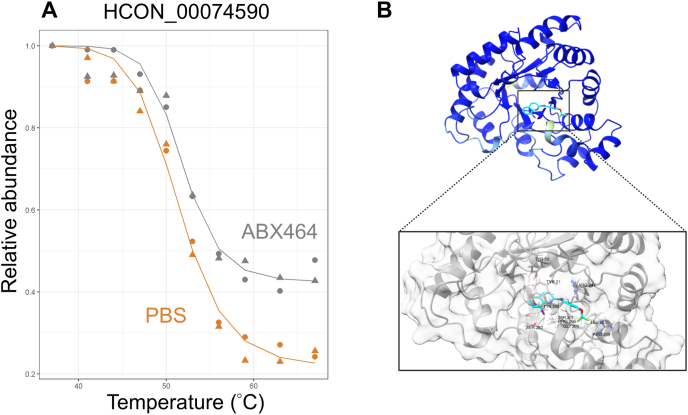
Thermal proteome profiling and *in silico* docking. (A) Thermal shift plot for the melting curves (temperature gradient 37 ^°^C–67 ^°^C) of a potential *Haemonchus contortus* protein target of ABX464, HCON_00074590. Data from two replicates. (B) The predicted interaction of the three-dimensional structure of HCON_00074590 (predicted using the algorithm Alphafold 2; UniProt accession number: A0A7I5E8V5) with ABX464 using the algorithm AutoDock Vina (displayed using ChimeraX software), with an inset showing the predicted binding pocket and associated residues. Carbon (grey), oxygen (red), nitrogen (blue), fluorine (green), chlorine (purple) and hydrogen (white) atoms are colour-coded.

Using this technique, we identified and quantified 4122 *H. contortus* proteins. A nonparametric analysis of the response curves (NPARC v 1.6.0, Childs et al., 2019) was then used to assess individual protein thermal profiles, and yielded 3357 melting profiles (Additional File 2). The melting profiles of statistically significant protein target candidates (Benjamini-Hochberg adjusted p-values (pAdj) < 0.05) were then plotted and manually inspected; only one protein, designated HCON_00074590 (Fig. 2A, Additional File 1: Table S1), predicted to be an aldo-keto reductase, was significantly stabilised in the presence of ABX464.

### 3.5 Possible binding sites of ABX464 to predicted protein structures identified *in silico*

Here, we modelled the three-dimensional structures of one protein, HCON_00074590, identified *via* TPP (cf. UniProt accession code: A0A7I5E8V5). As the protein model was confidently predicted (pLDDT >70), *in silico* docking of ABX464 with with protein structure was undertaken. We predicted the binding of ABX464 (Table 8) to this structure *in silico* (Additional File 3), and inferred that ABX464 binds to a cavity within this protein (10 surrounding residues; Vina −10.4; Fig. 2B) associated with the active site – six residues (Y21, Y200, S201, L203, G205 and P206), which are a part of the predicted catalytic tetrad active site, and one residue (Y50) as a proton donor active site.

**Table 8:** The AutoDock Vina scores and predicted binding cavities derived from *in silico-*docking of ABX464 with predicted protein structures (models 1 to 3).

Protein (Alphafold database identifier)	Model 1		Model 2		Model 3	
	Vina score (RMSD-ub)^a^	Predicted interacting residues (pLDDT >70)^b^	Vina score (RMSD-ub)^a^	Predicted interacting residues (pLDDT >70)^b^	Vina score (RMSD-ub)^a^	Predicted interacting residues (pLDDT >70)^b^
HCON_00074590 (A0A7I5E8V5)	−10.4 (0.0)	Y21, Y50, Y200, S201, L203, G205, P206, R209, R247, S283	−10.1 (7.767)	Y21, Y50, H112, Y200, S201, L203, G205, P206, R209, L245 R247	−10.1 (8.104)	Y21, Y50, H112, W113, N153, Y200, S201, G205, P206, L245 R247, S283, W284

a Root mean square distance upper bound, RMSD-ub.

b Predicted local distance difference test, pLDDT.

## 4 Discussion

Here, in an SAR investigation, we explored a pharmacophore surrounding ABX464's activity to inhibit the motility of *H. contortus* xL3s. In brief, it was inferred that hydrophobic, electron-withdrawing compounds at the *para* position of the aniline position were preferred. Substitutions at the aniline *meta* position were generally well-tolerated, including a phenyl moiety, implying that substitutions here may not be crucial to compound activity. However, in general, compounds with greater hydrophobicity did not reach the same MMI as their lower hydrophobicity counterpart, possibly indicating the need for a balance between hydrophobicity and activity. However, attempts at incorporating an endocyclic nitrogen moiety into the aniline structure did not reveal an active compound with lowered hydrophobicity; thus, more investigation is warranted to test this hypothesis. Substitutions at the *ortho* position of the aniline moiety were all associated with losses in activity. It was also determined that a halogen (fluoro, chloro or bromo) substitution at the 8-position on the quinoline moiety was associated with greater activity, whereas bulky, polar substitutions lost activity. Moving the position of the chloro functional group to the 3-position lead to a loss in activity, however, movements to the 4- or 6- position only slightly reduced activity. Of interest, a chloro substitution at the 5- position (**36**) found a slight increase in activity, whilst retaining an MMI of 100 %. Overall, future SAR analysis should focus on the continued exploration of the quinoline moiety, with the further aims of producing a potent compound with a reduced hydrophobicity.

ABX464 and one derivative, **36**, were also identified to inhibit *H. contortus* larval development and significantly inhibit motility of the adult female of *H. contortus*. In contrast, compounds **24** and **25**, which displayed motility and development inhibition on *H. contortus* larvae, did not significantly inhibit adult female motility. This information suggests that ABX464 (and derivatives) has multiple protein targets in different developmental stages, giving rise to differential anthelmintic activity. Alternatively, marked physiological, biochemical and molecular differences between larval and adult stages might also contribute to variability in drug pharmacokinetics (i.e. drug absorption, distribution, metabolism and/or excretion). Of note, both ABX464 and compound **36** were previously identified to have significant activity on *C. elegans* fourth-stage larvae (L4), suggesting the possibility for anthelmintic activity across several important nematodes (Shanley et al., 2024). As such, we further tested these compounds on larvae of *A. ceylanicum* and *N. americanus* (hookworms), *He. polygyrus, S. ratti* and adults of *T. muris*. ABX464 showed nematocidal activity against both *A. ceylanicum* and *S. ratti*, whereas compound **36** displayed significant activity against *He. polygyrus*. It was noted that, despite differences in activity between these compounds (which could be indicative of structural differences in a shared protein target between nematode species, or possibly reflective of these compounds binding to different targets), anthelmintic activity was displayed in multiple nematode species. Importantly, both ABX464 and compound **36** have been previously found to be non-cytotoxic and non-mitotoxic to human hepatoma (HepG2) cells (Shanley et al., 2024). Thus, the prospects for developing ABX464, and more potent derivatives, as a broad-spectrum anthelmintic might be favourable.

A significant barrier in anthelmintic development is the identification and understanding of drug-target interactions. As a step first step towards target deconvolution, we used TPP (Savitski et al., 2014; Mateus et al., 2020) to identify the possible drug-target interactions of ABX464 in *H. contortus* and identified one worm protein, HCON_00074590, which underwent compound-induced stabilisation. Subsequently, the predicted protein structure of HCON_00074590 was obtained from the AlphaFold2 database (Jumper et al., 2021). The *in silico–*prediction of ABX464 with HCON_00074590 was linked to a low Vina docking score, and revealed a binding pose within the predicted active site of HCON_00074590. Inferring function from the primary amino acid sequence (Doyle et al., 2020) and the predicted structure of HCON_00074590 (UniProt accession: A0A7I5E8V5) and from the related *C. elegans* orthologue, designated C35D10.6 (54 % identical, E value of 10^−108^), HCON_00074590 is predicted to belong to the aldo-keto reductase (AKR) superfamily of proteins. Although the complete function of HCON_00074590 has not been annotated, the general function of the AKR proteins is to reduce carbonyl substrates, playing an important role across a number of various species (reviewed by Penning, 2015). In human health, AKRs have been implicated in the pathogenesis of diabetes, bile acid deficiency and in retinoic acid signalling, becoming the focus of several drug development programs (Penning, 2015). It is possible that the interaction of ABX464 and HCON_00074590 disrupts one or more key biological functions, leading to the immobilisation and death of the parasite.

To explore whether ABX464 might target similar proteins across a number of nematode species, we compared the structure and function of HCON_00074590 to the proteins targets of ABX464 in *C. elegans*, as previously inferred (Shanley et al., 2024). Interestingly, HCON_00074590 differs in structure from the seven proteins in the free-living nematode, *C. elegans* (designated CRN-3, LUC-7L3, F30F8.9, CRN-3, RAGA-1, CDO-1, VPS-28 and DAB-1) proposed as targets of ABX464 (Shanley et al., 2024), and the putative target of ABX464 in humans, the cap binding complex (Campos et al., 2015; Vautrin et al., 2019; Tazi et al., 2020). Moreover, the *C. elegans* orthologue protein C35D10.6 was not identified *via* TPP as a compound that interacts with ABX464 (Shanley et al., 2024). It is plausible that the anthelmintic protein target is different between these two nematode species, due to the biological distinctiveness between the free-living and the parasitic worm. Although ABX464 also exhibited anthelmintic activity on *A. ceylanicum* and *S. ratti* larvae, it remains unclear whether a similar target is shared amongst parasite species, although orthologues of HCON_00074590 (*A. ceylanicum*, accession EYC36401, 72 % identity, E-value of 5 × 10^−162^, Schwarz et al., 2015; *S. ratti*, accession XP_024506708, 50 % identity, E value of 3 × 10^−95^, PRJNA304930) exist in both species. Of note, similar proteins exist in both *N. americanus* (accession XP_013293396.1, 70 % identity, E-value of 7 × 10^−163^) and *He. polygyrus* (accession VDP40277.1, 62 % identity, E-value of 5 × 10^−97^), yet appear not to exist in *T. muris*. Indeed, aldo-keto reductase proteins exist in humans, but have a low sequence identity with apparent homologues in *H. contortus* (accession NP_064695.3, 34 % identity, E-value of 1 × 10^−48^), suggesting possible selectivity for nematodes, if the aldo-keto reductase protein is a valid target of ABX464. Although promising as a tool TPP may have some limitations – for instance, it is possible that the primary target protein of ABX464 was not significantly denatured here under the present conditions, such that the actual nematode protein target of ABX464 was not identified. Thus, it is plausible that ABX464 does indeed target a protein (orthologue) shared among multiple nematode species, which was not identified *via* TPP, but could be identified by alternative methods. Thus, the TPP workflow could be enhanced through the complementary use of other methods, such as isothermal dose-response fingerprinting (Jafari et al., 2014), affinity chromatography (Ong et al., 2009) and/or photoaffinity labelling (Tulloch et al., 2017).

A genomics-focussed approach might also be utilised to identify and/or validate drug-protein interactions, through resistance-induction (Burns et al., 2006; Kaminsky et al., 2008), RNA interference (RNAi; Ashrafi et al., 2003; Hou et al., 2023) or CRISPR/Cas9 technology (Doudna and Charpentier, 2014). The identification of gene polymorphisms which confer drug resistance has been well-established in *C. elegans* (see Burns et al., 2006), and has been used to identify the targets of anthelmintics such as ivermectin (Dent et al., 1997) and monepantel (Kaminsky et al., 2008). Although a laborious and time-consuming process, resistance has also been successfully induced in *H. contortus via* repeated drug dosing (Kaminsky et al., 2008). Alternatively, RNAi-mediated gene knockdown has been performed in *C. elegans* (see Ashrafi et al., 2003), yet has been less successful in parasitic species such as *H. contortus* (reviewed by Hou et al., 2023). Another possible approach for target identification and/or validation would be the use of CRISPR/Cas9 genome engineering (Doudna and Charpentier, 2014), whereby genes can be knocked-out or knocked-in. Possibly, the effect of a small molecule on organisms in which a gene has been repressed, induced or deleted (*via* CRISPR) compared to a wild-type organism, could illuminate how anthelmintic activity is achieved. Although the latter approach has been used successfully in *C. elegans*, CRISPR/Cas9 technology has not yet been applied to *H. contortus*.

For the development of a new anthelmintic with a novel mechanism of action, it will be of vital importance to validate HCON_00074590 as a genuine *H. contortus* protein target *in vitro*. Following such a validation, a structure-guided drug design could be utilised to pursue an ABX464 analogue with activity comparable to other commercially available anthelmintics, such as monepantel or moxidectin. It would also be of interest to utilise another active compound, such as derivative **36**, in a TPP workflow on *H. contortus*, to establish whether ABX464 and **36** share the same predicted protein target(s). In an extension of this work, the antiparasitic activity of ABX464 should be evaluated *in vivo*, to assess whether nematocidal activity can be achieved within a representative animal host. Further evaluation of the biotransformation and ADME (absorption, distribution, metabolism, and excretion) properties of ABX464 would also be important for the development of a relatively broad-spectrum, efficacious anthelmintic compound.

## 5 Conclusion

The discovery of new anthelmintic compounds with novel mechanisms of action is a critical step towards tackling challenges associated with anthelmintic treatment failures and drug resistance in parasitic nematodes. Here, we used the model parasitic worm, *H. contortus*, to conduct early-stage antiparasitic drug discovery. We developed a nematocidal pharmacophore of ABX464 on *H. contortus* larvae and adults, identifying a derivative, **36**, which inhibited larval motility and development, and killed adults, at a greater than/equipotent activity than ABX464. Both ABX464 and compound **36** were subsequently tested against a panel of parasitic nematodes, with the results indicating low-to-moderate broad spectrum anthelmintic activity. Finally, as a first step towards identifying protein targets of ABX464 in a parasitic nematode, we used TPP and *in silico* simulations to evaluate drug-protein interactions of ABX464 in *H. contortus*. Future work should aim towards an extended SAR investigation of this compound in *H. contortus*, and focus on the validation of the drug targets proposed in this study. Overall, this work has provided the first steps towards developing a new anthelmintic.

## Ethics approval

This study was conducted in accordance with the institutional animal ethics guidelines (permit no. 1714374; The University of Melbourne).

## Availability of data and materials

All data generated or analysed during this study are included in this published article and its supplementary information files. The datasets presented in this study have been deposited in the PRIDE repository with the accession number PXD046553.

## Funding

We gratefully acknowledge financial support from the 10.13039/501100000923Australian Research Council (LP220200614 and LP180101085), PhylumTECH and Oz Omics Pty Ltd.

## Declaration of competing interest

The authors declare that they have no known competing financial interests or personal relationships that could have appeared to influence the work reported in this paper.
