# Supplementary material for: Structure-activity relationship and target investigation of 2-aryl quinolines with nematocidal activity
Source: Int J Parasitol Drugs Drug Resist. 2024 Jan 23;24:100522. doi: 10.1016/j.ijpddr.2024.100522 (PMC10845918; doi:10.1016/j.ijpddr.2024.100522)
Supplement: Multimedia component 1 [file mmc1.docx]

**SUPPLEMENTARY MATERIAL – ADDITIONAL FILE 1**

Structure-activity relationship and target investigation of 2-aryl quinolines with nematocidal activity

Harrison T. Shanley^a,b^, Aya C. Taki^a^, Nghi Nguyen^b^, Tao Wang^a^, Joseph J. Byrne^a^, Ching-Seng Ang^c^*,* Michael G. Leeming^c^, Shuai Nie^c^, Nicholas Williamson^c^, Yuanting Zheng^a^, Neil D. Young^a^, Pasi K. Korhonen^a^, Andreas Hofmann^a,d^, Bill C. H. Chang^a^, Tim N. C. Wells^e^, Cécile Häberli^f,g^, Jennifer Keiser^f,g^, Abdul Jabbar^a^, Brad E. Sleebs^a,b*^, Robin B. Gasser^a*^

^a^ *Department of Veterinary Biosciences, Melbourne Veterinary School, Faculty of Science, The University of Melbourne, Parkville, Victoria 3010, Australia*

^b^ *Chemical Biology Division,* *Walter and Eliza Hall Institute of Medical Research, Parkville, Victoria 3052, Australia*

^c^ *Melbourne Mass Spectrometry and Proteomics Facility, The Bio21 Molecular Science and Biotechnology Institute, The University of Melbourne, Parkville, Victoria 3010, Australia*

^d^*National Reference Centre for Authentic Food, Max Rubner-Institut, 95326 Kulmbach, Germany*

^e^ *Medicines for Malaria Venture (MMV), 1215 Geneva, Switzerland*

^f^ *Medical Parasitology and Infection Biology, Swiss Tropical and Public Health Institute, 4123 Allschwil, Switzerland*

^g^ *University of Basel, 4001 Basel, Switzerland*

_______

* Corresponding authors. Chemical Biology Division, Walter and Eliza Hall Institute of Medical Research, Parkville, Victoria 3052, Australia; Department of Veterinary Biosciences, Melbourne Veterinary School, Faculty of Science, The University of Melbourne, Parkville, Victoria 3010, Australia.

*E-mail addresses:* [robinbg@unimelb.edu.au](mailto:robinbg@unimelb.edu.au) (R.B. Gasser); [sleebs@wehi.edu.au](mailto:sleebs@wehi.edu.au) (B.E. Sleebs).

**Index**

Page

2 Figure S1 ABX464 and derivatives chemical names (IUPAC) and structures

8 Figure S2 *H. contortus* larvae dose response curves

9 Figure S3 *H. contortus* larvae development inhibition

10 Figure S4 *H. contortus* adult female motility inhibition

11 Table S1 TPP statistics summary

**Supplementary Figures 1.** The chemical structures and names (IUPAC nomenclature) of ABX464 and derivatives.

*8-Chloro-N-(4-(trifluoromethoxy)phenyl)quinoline-2-amine (****1****)*.

*8-Chloro-N-phenylquinolin-2-amine (****2****).*

*4-((8-Chloroquinolin-2-yl)amino)benzonitrile (****3****).*

*8-Chloro-N-(4-methoxyphenyl)quinoline-2-amine (****4****).*

*8-Chloro-N-(p-tolyl)quinolin-2-amine (****5****)*.

*8-Chloro-N-(4-chlorophenyl)quinolin-2-amine (****6****)*.

*8-Chloro-N-(4-fluorophenyl)quinolin-2-amine (****7****)*.

*N-([1,1’-Biphenyl]-4-yl)-8-chloroquinolin-2-amine (****8****).*

*8-Chloro-N-((3-trifluoromethoxy)phenyl)quinolin-2-amine (***9***).*

*8-Chloro-N-(3-(trifluoromethyl)phenyl)quinolin-2-amine (****10****).*

*3-((8-Chloroquinolin-2-yl)amino)benzonitrile (****11****).*

*8-Chloro-N-(3-methoxyphenyl)quinolin-2-amine (****12****).*

*8-Chloro-N-(m-tolyl)quinolin-2-amine (****13****).*

*8-Chloro-N-(3-chlorophenyl)quinolin-2-amine (****14****).*

*8-Chloro-N-(3-fluorophenyl)quinolin-2-amine (****15****).*

*N-([1,1’-Biphenyl]-3-yl)-8-chloroquinolin-2-amine (****16****).*

*8-Chloro-N-(2-chlorophenyl)quinolin-2-amine (****17****).*

*8-Chloro-N-(2-methoxyphenyl)quinolin-2-amine (****18****).*

*8-Chloro-N-(2-fluorophenyl)quinolin-2-amine (****19****).*

*2-((8-Chloroquinolin-2-yl)amino)benzonitrile (****20****).*

*8-Chloro-N-[6-(trifluoromethyl)-3-pyridyl]quinolin-2-amine (****21****).*

*8-Chloro-N-(3-chloro-4(trifluoromethoxy)phenyl)quinoline-2-amine (****22****).*

*8-Chloro-N-(4-chloro-3(trifluoromethyl)phenyl)quinoline-2-amine (****23****)*.

*8-Chloro-N-(3,4-dichlorophenyl)quinolin-2-amine (****24****).*

*8-Chloro-N-(4-(trifluoromethyl)phenyl)quinolin-2-amine (****25****).*

*8-Chloro-N-(2-(trifluoromethoxy)phenyl)quinolin-2-amine (****26****).*

*N-([1,1’-Biphenyl]-2-yl)-8-chloroquinolin-2-amine (****27****).*

*8-Chloro-N-(2-(trifluoromethyl)phenyl)quinolin-2-amine (****28****).*

*8-Chloro-N-methyl-N-(4-(trifluoromethoxy)phenyl)quinolin-2-amine (****29****).*

*8-Chloro-N-[5-(trifluoromethyl)-2-pyridyl]quinolin-2-amine (****30****).*

*N-(3,4-Bis(trifluoromethyl)phenyl)-8-chloroquinolin-2-amine (****31****).*

*8-Chloro-2-(4- (trifluoromethoxy)phenoxy)quinoline (****32****).*

*4-((8-Chloroquinolin-2-yl)amino)phenol (****33****).*

*N-(4-(Trifluoromethoxy)phenyl)quinoline-2-amine (****34****).*

*6-Chloro-N-(4-(trifluoromethoxy)phenyl)quinolin-2-amine (****35****)*.

*5-Chloro-N-(4-(trifluoromethoxy)phenyl)quinolin-2-amine (****36****).*

*4-Chloro-N-(4-(trifluoromethoxy)phenyl)quinolin-2-amine (****37****).*

*3-Chloro-N-(4-(trifluoromethoxy)phenyl)quinolin-2-amine (****38****).*

*8-Methoxy-N-((4-trifluoromethoxy)phenyl)quinolin-2-amine* *(****39****).*

*8-Methyl-N-((4-trifluoromethoxy)phenyl)quinolin-2-amine (****40****).*

*8-Fluoro-N-((4-trifluoromethoxy)phenyl)quinolin-2-amine (****41****).*

*8-Bromo-N-((4-trifluoromethoxy)phenyl)quinolin-2-amine (****42****).*

*8-Phenyl-N-((4-trifluoromethoxy)phenyl)quinolin-2-amine (****43****).*

*2-((4- (Trifluoromethoxy)phenyl)amino)quinoline-8-carbonitrile (****44****).*

*N-(4-(Trifluoromethoxy)phenyl)pyridin-2-amine (****45****)*.

**Supplementary Figure 2.** The potencies of ABX464 and three active derivative compounds (**24**, **25** and **36**) against exsheathed third-stage larvae of *Haemonchus contortus* with reference to one control compound (monepantel). The dose-response curve shows the reduction of *H. contortus* motility at 168 h. Data points represent three independent experiments conducted in triplicate; the mean ± the standard error of the mean (SEM).

******

**Supplementary Figure 3.** The potencies of ABX464 and three active derivative compounds (**24**, **25** and **36**) against larvae of *Haemonchus contortus* with reference to one control compound (monepantel). The dose-response curve shows the inhibition of *H. contortus* larval development at 168 h. Data points represent three independent experiments conducted in triplicate; the mean ± the standard error of the mean (SEM).

******

******

**Supplementary Figure 4.** The *in vitro* motility inhibition (%) of ABX464 and three active derivative compounds (**24**, **25** and **36**) against adult females of *Haemonchus contortus*, with reference to two control compounds (monepantel and moxidectin). Motility scores (assessed at 3-, 6-, 12- and 24-h time points) for each compound were calculated and normalised to a negative control (100 % motility), and were recorded as a percentage. Data points represent one experiments conducted in triplicate; the mean ± standard deviation (SD

**Supplementary Table 1.** The p-values, adjusted p-values and F-statistics of protein HCON_00074590 identified in a thermal proteome profiling assay with ABX464.

| **Protein** | **p-Value** | **p-Value (Benjamini-Hochberg-adjusted)** | **F-statistic** |
| --- | --- | --- | --- |
| HCON_00074590 | 5.68e-6 | 0.006 | 24.62 |
